# Acute elevations in serum hormones are attenuated after chronic training with traditional isoinertial but not accentuated eccentric loads in strength‐trained men

**DOI:** 10.14814/phy2.13241

**Published:** 2017-04-10

**Authors:** Simon Walker, Keijo Häkkinen, Guy Gregory Haff, Anthony J. Blazevich, Robert U. Newton

**Affiliations:** ^1^ Faculty of Sport and Health Science and Neuromuscular Research CenterUniversity of JyväskyläJyväskyläFinland; ^2^ Centre for Exercise and Sports Science Research (CESSR)Edith Cowan UniversityJoondalupAustralia; ^3^ Institute of Human PerformanceThe University of Hong KongHong KongChina

**Keywords:** Hypertrophy, overload, progression, resistance

## Abstract

It has been proposed that the maintenance of acute hormonal responses reveal an efficacy of a training stimulus to evoke ongoing increases in strength and muscle mass. We previously observed that maximum strength continued to improve throughout a 10‐week period in an accentuated eccentric loading group (AEL) but not a traditional isoinertial loading (ISO) group. Therefore, this study investigated whether the magnitude of acute hormonal responses was greater (i.e., maintained) in AEL compared to ISO at the end of the training period. Subjects in AEL (eccentric load = concentric load + 40%) and ISO performed experimental loading tests (three sets of 10 repetitions in the leg press and knee extension exercises) during weeks 2 and 9 of the training period. Blood samples collected during these experimental loadings were analyzed for serum testosterone, growth hormone and cortisol concentrations. Maximum isometric knee extension torque (MVC) and lower‐limb lean mass were assessed before and after 5 and 10 weeks of training. Acute testosterone, growth hormone and cortisol responses to traditional isoinertial loading were reduced at the end of the training period but were not reduced after accentuated eccentric load training (*P* < 0.05‒0.1 between‐groups). Increases in MVC and lower‐limb lean mass over weeks 6‒10 were greater in AEL compared to ISO (MVC: 7.3 ± 5.4 vs. −0.4 ± 7.2%, *P* = 0.026 for between‐group difference; lower‐limb lean mass: 1.6 ± 2.2 vs. −0.2 ± 1.4%, *P* = 0.063 for between‐group difference). The maintenance of acute hormonal responses and continued strength gain in AEL but not ISO are consistent with the hypothesis that maintained acute responses indicate an efficacy of a training stimulus to evoke ongoing adaptation. However, since relationships between hormonal responses and training‐induced adaptations were not statistically significant, the data suggest that tracking of acute hormonal responses on an individual level may not provide a sensitive enough guide for decisions regarding program design and periodization.

## Introduction

Hormones that regulate the anabolic/catabolic environment are a primary target for manipulation given that their presence has diverse, largely positive, effects on multiple tissues (Cuneo et al. 1991; Bhasin et al. 1996; Florini et al. 1996). In addition to direct muscular and venous injection, a large body of research has examined the potential for exercise to stimulate hormonal release and, thus, contribute to tissue adaptation. With regard to strength training, medium‐load high‐volume protocols with relatively short inter‐set rest intervals, favored by bodybuilders, produce the greatest acute elevations in serum testosterone (T), growth hormone (GH) and cortisol (C) concentrations (e.g., Kraemer et al. 1990; Häkkinen and Pakarinen 1993). These acute elevations could be a result of greater production and secretion into circulation or reduced uptake by target tissues through their receptors or a combination of both events. Nevertheless, relationships between the acute elevation in T, GH, and C during a single training session and long‐term increases in primary outcome measures such as strength and muscle mass have rarely been observed. Consequently, there is controversy as to whether such acute hormonal elevations induce, or even contribute to, outcomes such as muscle hypertrophy (Schroeder et al. 2013).

Several possible reasons for a lack of a direct relationship between acute hormonal responses and specific adaptations include that: (1) transient loading‐induced elevations are not involved in muscular adaptive processes (e.g., West et al. 2010; Wilkinson et al. 2006), (2) acute hormonal responses exceed the minimum required level for tissue remodeling (i.e., only a small elevation in concentration is needed to stimulate processes that lead to adaptation) or, alternatively, are far below the required level to directly influence adaptation (e.g., compared to exogenous administration), (3) acute responses primarily affect tissues other than muscle, or affect non‐contractile collagen but not myofibrillar protein synthesis (e.g., Doessing et al. (2010), (4) the transient elevation reflects a response proportional to the level of disturbed homeostasis during the session (e.g., reduced T clearance rates (Ahtiainen et al. 2015)) and/or the level of substrate breakdown/consumption (Simmons et al. 1984; Yarasheski et al. 1992) and thus indicates a stress dose‐response association, and (5) acute hormonal responses to a nonchanging exercise stimulus are altered (i.e., decreased) as the training program or level of adaptation progresses, so longer‐term effects are attenuated.

With respect to reasons 4 and 5 in particular, previous studies in untrained young individuals have reported greater acute testosterone and growth hormone responses after short heavy‐resistance training periods (Kraemer et al. 1999; Ahtiainen et al. 2003; Izquierdo et al. 2009; Walker et al. 2015). Also, a short period of training appears to induce larger acute hormonal responses in untrained elderly individuals who exhibit a low pre‐training response (Kraemer et al. 1999; Häkkinen et al. 2001, 2002). Conversely, however, the acute response seems to be substantially reduced during repeated training sessions in already well‐trained individuals (Ahtiainen et al. 2003; Mangine et al. 2015). This is an interesting observation since it is clear that adaptation to the same training program is more robust and prolonged in previously untrained individuals, and that submaximal loading leads to a lower magnitude of acute hormonal responses (e.g., Raastad et al. 2000). Consequently, it may be prudent to assess acute hormone responses during several training sessions throughout a training period to determine whether training‐induced adaptations in strength or muscle mass are associated with changes in acute hormone responses. It should be stressed that possible associations do not infer direct cause and effect, but rather it has been proposed that a continued acute hormonal response, possibly evoked by homeostatic disturbance (as mentioned above), may reflect the efficacy of that particular training stimulus to further elicit increases in strength and muscle mass (Walker et al. 2015). Hence, a reduced hormonal response may indicate a need to alter the training program in a systematic way in order to augment the adaptive potential, that is, periodization strategies need to be used.

In order to provide a greater stimulus, and thus maximize long‐term adaptation, a number of training strategies can be utilized by athletes, including the use of accentuated eccentric loads. Accentuated eccentric loading is a strength training method that utilizes the force reserve available during the eccentric phase (Kats 1939) through imposition of a greater external load during the eccentric phase (i.e., typically greater than the concentric maximum load) and then a submaximal load during the concentric phase. Few studies have investigated the acute hormonal responses to accentuated eccentric loading versus traditional isoinertial loading, however similar acute responses to training have been observed in untrained individuals (Yarrow et al. 2007, 2008). Nonetheless, no study has examined the responses in strength‐trained individuals, or determined whether the additional loading can elicit a larger, ongoing acute response during a training period.

Some experimental evidence suggests that accentuated eccentric loading may lead to greater gains in strength and muscle mass in strength‐trained athletes than isoinertial loading (Brandenburg and Docherty 2002; Friedmann‐Bette et al. 2010). This divergent response may require some time to become apparent, as our recently published data in strength‐trained individuals (Walker et al. 2016) showed similar gains in strength and muscle mass during an initial 5‐week training period, but then those using accentuated eccentric loads uniquely continued to increase their strength during a subsequent 5‐week training period. This provided us a unique opportunity to analyze blood samples collected during that study to test the hypothesis that a greater or maintained acute hormonal response at the end of a training period would be observed in those training with accentuated eccentric loads. Conversely, this setting also allowed us to assess whether the lack of adaptation in the group training with isoinertial loads would be paralleled by a lower acute hormonal response, as previously observed by Ahtiainen et al. (2003) and Mangine et al. (2015). That is, the present study investigated whether acute hormonal responses can indicate whether a training program still creates homeostatic disturbance and stimulates ongoing adaptive changes.

## Materials and Methods

### Study design

The study design and testing methods have been described in our recent publication (Walker et al. 2016). Subjects underwent background screening and a familiarization session regarding the test procedures of the study. Three to 4 days after the familiarization session the subjects reported to the laboratory in a fasted state (10 h) and had whole‐body composition assessed by Dual‐energy X‐ray Absorptiometry (DXA), and after another 3‒4 days they completed maximal unilateral isometric knee extension tests. The subjects were then matched in threes based on body mass and knee extensor strength and randomly assigned to either the isoinertial (ISO) or accentuated eccentric load (AEL) strength training groups or control group. In this arm of the study, blood samples were collected from ISO and AEL. The body composition and strength test sessions were repeated after 5 (mid‐training) and 10 (post‐training) weeks of training. During the mid‐ and post‐training tests, the DXA measurements were performed 3–4 day after the last training session and subsequent strength tests were conducted after a further 3 day, so that strength tests took place 6‒8 day after the last training session.

Once all pre‐training tests were completed, the subjects engaged in a 10‐week training period for the lower limbs, consisting of three sets of bilateral leg press, three sets of unilateral knee extension and three sets of bilateral knee flexion as described previously (Walker et al. 2016). Training was performed twice per week with at least 48 h recovery between training sessions. The subjects performed sets of six repetition maximum (6‐RM) in training session 1 and sets of 10‐RM in training session 2 of each week. Each subject had their training and testing times standardized throughout the study (± 1 h).

**Figure 1:**
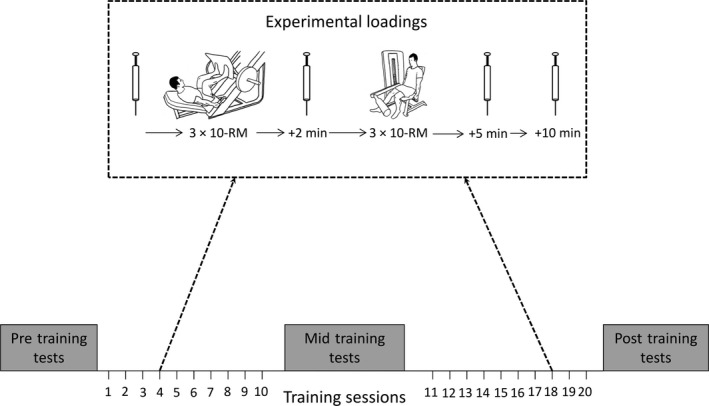
Experimental loading protocol integrated into the subjects' 10‐week training intervention. Tests were performed during sessions 4 and 18 (as depicted) and blood samples were obtained immediately before and 2 min after the leg press exercise, as well as 5 and 15 min after the unilateral knee extension exercise.

Acute responses to the experimental loading (see below) were assessed during the second (i.e. after 1 week of training familiarization) and ninth weeks of the 10‐week training period (see Fig. 1). In these testing sessions the subjects performed 10‐RM training as prescribed in their training program, however, alcohol (48 h) and caffeine (6 h) intake was restricted and dietary intake (12 h pre‐loading) was replicated before both loading tests in order to standardize their pre‐loading nutrition. Blood samples were obtained immediately pre‐loading, 2 min after the leg press sets (i.e., mid‐loading) and 5 min (post‐loading) and 15 min (post‐loading 15) after the knee extension sets.

## Subjects

A sub‐sample from our recent publication (Walker et al. 2016) consisting of 18 healthy young men participated in this arm of the study after being informed of all the risks and providing signed informed consent. The subjects had a strength training background (2.7 ± 2.3 year; initial 10‐RM inclined leg press load = 2.1 ± 0.5 kg:BM in ISO and = 1.7 ± 0.3 kg:BM in AEL). The study methods were approved by the ethics committee of Edith Cowan University and conducted according to the Declaration of Helsinki.

There were no differences in the baseline characteristics of subjects in ISO (age 21 ± 2 year, height 1.78 ± 0.08 m, body mass 79 ± 12 kg, *n* = 9) or AEL (age 21 ± 3 year, height 1.8 ± 0.07 m, body mass 76 ± 11 kg, *n* = 9). Subjects were instructed to continue with their normal upper body strength training program with this study being the only strength training of the legs performed during the 10‐week period, however upper body training could not take place within 24 h of the experimental loadings in weeks 2 and 9. The subjects completed training diaries to log the exercise performed outside of the study. According to these diaries, subjects participated in noncompetitive recreational activities at a frequency of 1‒3 times per week; seven subjects participated in ball sports, three participated in martial arts, two subjects cycled, one subject jogged and one swam (ISO: *n* = 6, AEL: *n* = 8), while the others only performed upper body strength training external to the present intervention. One subject from AEL was ill during week 9 and did not complete the testing; therefore, the final sample size for AEL was 8.

### Experimental loading procedures

The subjects performed acute experimental loading tests during weeks 2 and 9 of the 10‐week training period; week 1 allowed the subjects to become familiar with accentuated eccentric load training while week 10 acted as a taper week before the post‐training tests. The loading protocol consisted of three sets of 10‐RM of the bilateral leg press exercise (45° leg press, Cybex international Inc, Medway, USA) followed by three sets of 10‐RM unilateral knee extensions (VR3 leg extension, Cybex international Inc, Medway, USA). This was identical in sets, repetitions and actions performed during the strength training intervention, with the exception that the knee flexion exercise was excluded from the testing. A 2‐min inter‐set rest interval was given between leg press sets and a 1‐min rest interval was given between knee extension sets (which matched the rest periods used in training).

ISO performed the exercises with the same load for both concentric and eccentric phases, while AEL performed the exercises with 40% greater load during the eccentric phase compared to the concentric phase (i.e., eccentric load = concentric load + 40%). Custom weight‐releasers were used to add the additional eccentric load to the leg press exercise while weight plates were manually added and removed by the training supervisor(s) with the use of a custom‐built pin for the knee extension exercise. Both groups performed the concentric and eccentric phases of the lift with a 2:2 sec tempo (i.e., 4 sec in total), which was monitored by the investigator. All exercises were performed from an approximately knee angle of 85° and a maximum extension of 175° knee angle (180° = full extension). To ensure this range of motion was achieved, individual markings were placed onto the leg press device for ISO and the height of the weight‐releasers was set individually for AEL. For the knee extension exercise, the subjects were instructed to descend until the weight‐stack plates touched and then extend until the lever arm made contact with a rubber stopper that was positioned individually for each subject. Each subject performed 10 repetitions in all sets, however light assistance was provided by the investigator and the load was reduced for the following set if a subject could not voluntarily complete 10 repetitions. The total load (concentric load+eccentric load) and number of repetitions that were performed by each subject were recorded and used for analysis (i.e. volume load). Only a small amount of water (<200 mL) was allowed ad libitum during the experimental loadings.

Subjects remained seated throughout the rest periods as well as the blood sampling periods during the loading test. Blood samples were taken (from the antecubital vein and fingertip) immediately before loading, 2 min after the leg press sets, and 5 min and 15 min after the knee extension sets. Venous blood samples were collected into heparinized serum separator tubes (8.5 mL Venosafe SST 2 advance, Becton Dickinson and Co. vacutainer, Plymouth, UK), which stood at room temperature for 15 min before being centrifuged (5702R centrifuge, Eppendorf AG, Hamburg, Germany) for 10 min at 3 500 rpm. The serum was pipetted into 1.5 mL tubes and stored at −80°C until further analysis. Total testosterone (T), 22 kDa growth hormone (GH) and cortisol (C) were analyzed using chemiluminescence techniques (Immulite 1000, Siemens, Illinois, USA). Assay sensitivities for these hormones were: T = 0.5 nmol.L^−1^, 22 kDa GH = 0.01 mg·L^−1^, C = 5.5 nmol·L^−1^, with intra‐assay reliability (coefficient of variation %) of T = 5.7%, 22 kDa GH = 5.8%, C = 7.9%. Each subject's samples were analyzed in the same run.

Fingertip blood samples were collected onto LactatePro strips (Arkray Inc, Kyoto, Japan), into HemoCue microcruvettes (HemoCue 201, HemoCue AB, Ängelholm, Sweden) and into non‐heparinized capillary tubes (32 *μ*L micro‐capillary tubes, Selzer GmbH, Waghäusel, Germany) and used to analyze blood lactate concentration, hemoglobin and hematocrit, respectively. The manufacturer's instructions were followed for blood lactate and hemoglobin analysis, which were performed immediately. To measure hematocrit level, the capillary tubes were plugged with plasticine, centrifuged at 14,328*g* for 5 min (MPW‐212 centrifuge, MPW medical instruments, Poland) and the serum: red blood cell ratio determined. Hemoglobin analysis was performed in duplicate and hematocrit in triplicate with the mean value recorded for further analysis (CV% = 3.2% and 1.9%, respectively). Hemoglobin and hematocrit values were then used to assess plasma volume changes using the equation of Dill and Costill (1974) (approximately 6% during all loadings). Since there were no differences in the acute plasma volume change between groups, uncorrected serum hormone concentrations are presented.

### Strength training procedures

Lower limb strength training was completed twice per week with at least 48 h recovery between sessions (i.e., Monday and Thursday or Tuesday and Friday). The exercises performed during training were the bilateral leg press (45° leg press, Cybex international Inc, Medway, USA) and unilateral knee extension (VR3 leg extension, Cybex international Inc, Medway, USA). Isoinertial bilateral knee flexion (seated leg curl, PulseStar fitness, Cheshire, UK) exercise was performed by both groups at the completion of training to maintain antagonist muscle strength. During the first session of the week, subjects performed three sets of 6‐repetition maximum (6‐RM) for each exercise and during the second session of the week performed three sets of 10‐RM for each exercise. This loading paradigm was chosen to induce improvements in maximum strength and muscle mass. During each training session, both groups performed the last set as a true repetition maximum (i.e., to, or very close to, concentric failure), which ensured progression and allowed improvements to be monitored. Therefore, the loads were individualized so that the effect of the additional 40% eccentric load in AEL still led to concentric failure at the required sixth or tenth repetition, respectively. A 2‐min inter‐set rest interval was given for the leg press exercise and a 1‐min inter‐set rest interval was given for the knee extension and flexion exercises. After each training session the subjects were provided with a nutrient drink composed of 23 g protein (containing 1.95 g leucine), 3 g carbohydrate and 1.6 g fat (Total+ Vital Strength, Power Foods International Plc, Marrickville, Australia) in order to standardize post‐training nutrition between the groups. During the experimental loading tests, this drink was provided after the final blood sample.

### Body composition measurements

Whole‐body composition and lower‐limb segmental mass were assessed using DXA (Hologic Discovery A, Waltham, WA). Before scanning, the subjects were positioned supine on the scanning bed with both arms pronated by their side and hands facing the table. The researcher manually assisted subjects to straighten their head, torso and pelvis, internally rotate and fixate their legs and feet at 45°, and ensure they were located within the DXA scanning zone. This has been shown to produce a scan/re‐scan coefficient of variation below 1% in our laboratory (Peiffer et al. 2010). Whole‐body scans were analyzed using the inbuilt analysis software (Version 12.4; QDR for Windows, Hologic, Waltham, WA). The predefined whole‐body model was applied to the scan image, separating the body into axial and appendicular sections to determine whole‐body composition and segmental composition of the legs. The same researcher performed all measurements and analyses.

### Isometric knee extension tests

Subjects were positioned into a custom‐built isometric dynamometer (Edith Cowan University, Joondalup, Australia). The knee and hip angles were set to 110° and 100°, respectively, and the subjects were secured firmly by inelastic straps across the shoulders, hips, and ankle (straight leg and full knee extension = 180°). The subjects performed unilateral isometric knee extension trials “as fast and then as hard as possible” and maintained the peak torque for approximately 3–4 sec. Three trials were performed, with a fourth trial being required if the third trial yielded more than 5% greater torque compared to the previous trials. Real‐time visual feedback was provided to instruct the subject to rapidly achieve maximum torque and maintain the contraction for 3–4 sec. Loud, verbal encouragement was given throughout each trial to ensure maximum effort. Torque data were sampled at 2000 Hz and filtered offline (20 Hz low‐pass, 4th‐order zero‐lag Butterworth). Analysis was performed offline and assessed for maximum torque. Coefficient of variation % for isometric torque was 4.1%.

### Statistical analyses

Standard methods were used to calculate means and standard deviations. Based on sample size and data distribution considerations, significant main effects were analyzed by Friedman's test for multiple comparisons and Wilcoxon matched pairs were used for posthoc tests. Relative changes over time (Δ%) were assessed by Mann–Whitney U test. Effect sizes for between‐group differences were calculated using Hedge's *g* (defined as small; <0.3, medium; 0.3–0.8, and large; >0.8) with corresponding 95% confidence intervals (95%CI). Correlations were performed with Spearman's rank correlation coefficient and CI's calculated using Fisher's r‐to‐z transformation. The level of significance was set at 0.05.

## Results

**Figure 2:**
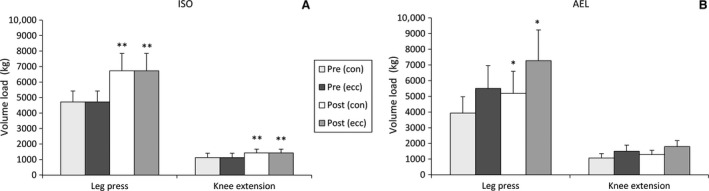
Volume load (mean ± SD) separated into each phase during the leg press and knee extension exercises in the isoinertial training group (A) and accentuated eccentric load group (B) in week 2 (“Pre‐training”) and week 9 (“Post‐training”) loadings. *P < 0.05 versus pre‐training, **P < 0.01 versus pre‐training. con = concentric, ecc = eccentric phase.

The volume load lifted during the experimental loadings increased significantly in both groups from week 2 (i.e., pre‐training) to week 9 (i.e., post‐training) (*P* < 0.05, Fig. 2A and B). There were no between‐group differences at either time point or in the change from week 2 to 9.

**Figure 3:**
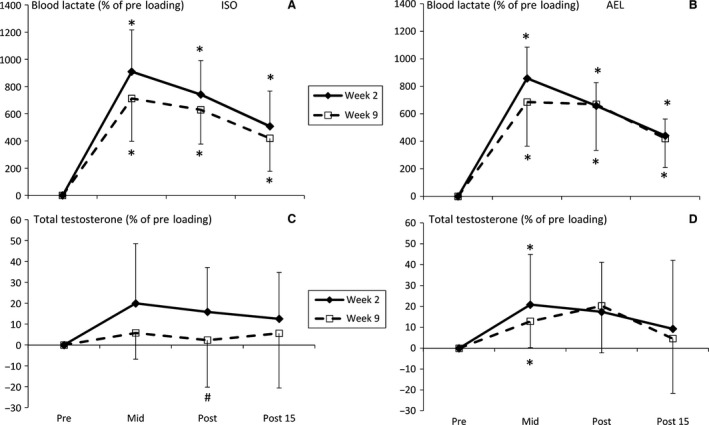
Relative changes (mean ± SD) in blood lactate (A: ISO, B: AEL) and total testosterone (C: ISO, D: AEL) concentrations during loadings in weeks 2 and 9 of the 10‐week training period. *P < 0.05 versus pre‐loading, #*P* < 0.05 versus corresponding time point in AEL.

There were no significant differences in pre‐loading (i.e., resting) hormone concentrations from weeks 2 to week 9 in either group (Table 1). Experimental loading evoked significant elevations in blood lactate concentration in both groups at weeks 2 and 9 at all time points, as shown in Figure 3. Elevations in T were observed at mid‐loading (i.e., after the leg press exercise; *P* < 0.05) and a trend toward an elevation at post‐loading (i.e. at the end of the training session; *P* = 0.053) was observed in AEL during weeks 2 and 9 (Fig. 3C and D). There was a significant between‐group difference in T response measured at post‐loading during week 9 (*P* = 0.05, *g *=* *0.94, 95%CI = −0.06–1.94, Fig. 3C) where no acute change in T concentration was observed in ISO.

**Table 1:** Pre‐loading serum hormone concentrations in weeks 2 and 9 of the 10‐week training period (mean ± SD)

	Week 2		Week 9	
	ISO	AEL	ISO	AEL
Lactate (mmol/L)	1.2 ± 0.4	1.3 ± 0.4	1.6 ± 0.6	1.8 ± 0.9^a^
Testosterone (nmol/L)	12.3 ± 4.3	14.1 ± 5.7	12.0 ± 3.9	15.4 ± 4.7
Cortisol (nmol/L)	290 ± 120	307 ± 53	324 ± 114	352 ± 102
22 kDa GH (μg/L)	0.2 ± 0.3	0.3 ± 0.4	0.9 ± 1.1	0.3 ± 0.5

GH, growth hormone.

a *P* < 0.05 versus week 2 (“pre‐training”).

**Figure 4:**
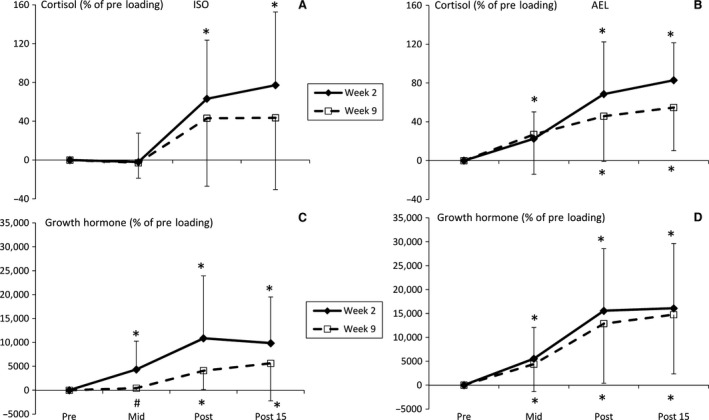
Relative changes (mean ± SD) in cortisol (A: ISO, B: AEL) and 22 kDa growth hormone (C: ISO, D: AEL) concentrations during loadings in weeks 2 and 9 of the 10‐week training period. *P < 0.05 versus pre‐loading, #*P* < 0.05 versus corresponding time point in AEL.

In AEL, serum cortisol concentrations were elevated at mid‐, post‐, and 15 min post‐loading in week 2 and at post‐ and 15 min post‐loading in week 9. In ISO, cortisol concentrations were elevated at post‐ and 15 min post‐loading in week 2 (*P* < 0.05, Fig. 4A and B) but no changes were observed in week 9. GH was significantly elevated in weeks 2 and 9 at all time points in AEL, and in ISO GH concentrations were significantly elevated at all time points in week 2 (*P* < 0.05) and also at post‐ and 15 min post‐loading in week 9. The mid‐loading GH response in ISO during week 9 was significantly lower than AEL (*P* = 0.05, *g *=* *0.95, CI = −0.06–1.95, Fig. 4C). There were also near‐significant between‐group differences in C at mid‐loading (*P* = 0.063, *g *=* *0.93, CI = −0.07–1.93) and GH at post‐ (*P* = 0.063, *g *=* *0.92, CI = −0.08–1.93) and 15 min post‐loading (*P* = 0.087, *g *=* *0.84, 95%CI = −0.15–1.84) during week 9.

**Figure 5:**
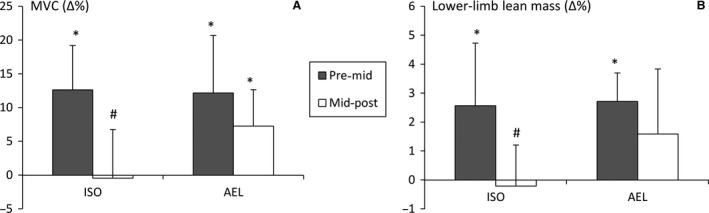
Relative improvements (mean ± SD) in maximum isometric knee extension torque (A) and lower‐limb lean mass (B) from pre‐ to mid‐training and mid‐ to post‐training. *P < 0.05 within group, #*P* < 0.05 between groups.

For the subjects included in this hormone analysis arm of the study, increases in maximum knee extension torque (ISO: 12.6 ± 6.5%, *P* < 0.05; AEL: 12.2 ± 8.5%, *P* < 0.05) and lower‐limb lean mass (ISO: 2.6 ± 2.2%, *P* < 0.05; AEL: 2.7 ± 1.0%, *P* < 0.05) were statistically significant at mid‐training in both groups. Thereafter, only AEL significantly increased torque (7.3 ± 5.4%, *P* < 0.05, Fig. 1A) and the relative increases in knee extension torque and lower‐limb lean mass were greater in AEL compared to ISO from mid‐ to post‐training (*P* = 0.026, *g *=* *1.14, 95%CI = 0.11–2.16 and *P* = 0.06, *g *=* *0.94, 95% CI = −0.06–1.94, Fig. 5A and B, respectively).

**Figure 6:**
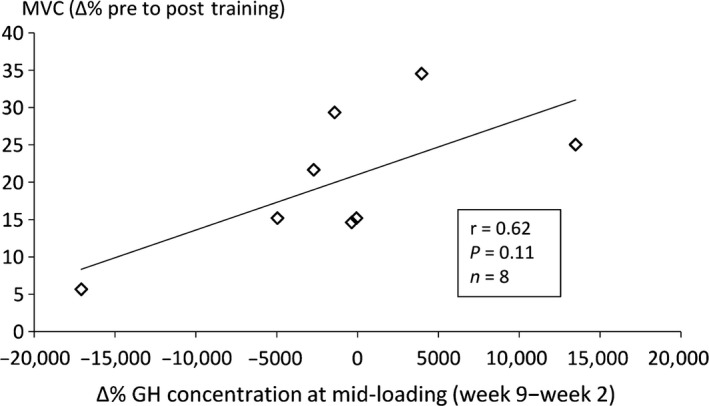
Relationship between training‐induced increases in maximum isometric knee extension torque (MVC) and the change in acute GH response from week 2 to week 9 at mid‐loading in AEL.

In AEL, correlation analysis revealed moderate but non‐significant positive relationships between increases in MVC and lower‐limb lean mass and the changes in the acute GH response throughout loading (i.e. week 9 concentration−week 2 concentration). For example, the change in acute GH response at mid‐loading was related to an increased MVC (*r* = 0.62, *P* = 0.11, 95% CI = −0.15–0.92, *n* = 8, Fig. 6) and lower‐limb lean mass (*r* = 0.53, *P* = 0.16, 95% CI = −0.28–0.90, *n* = 8) pre‐ to post‐training.

## Discussion

In this study, a traditional isoinertial (10‐RM) lower limb strength training session elicited similar acute testosterone (T), growth hormone (GH) and cortisol (C) responses as a training session performed with accentuated eccentric loading in already strength‐trained individuals. However, an attenuation of these loading‐induced responses was observed in ISO after 9 weeks of training whilst these responses were maintained in AEL. While similar improvements in strength and lean mass might have been expected during weeks 0‒5, given the new training stimulus, spotting, external motivation, and standardized post‐workout nutrition, weeks 6–10 would likely identify whether AEL provides a prolonged stimulus for adaptation. The differential response in strength and lean mass increases during weeks 6–10 that occurred were in parallel to the maintained acute hormonal response. However, no statistically significant correlations were observed between increases in strength or lower‐limb lean mass and the changes in acute hormonal responses over the training period. Therefore, it was not the case that greater acute (absolute) responses in individuals after the training period were associated with greater adaptive response.

It should be stressed that the increases in strength and lower‐limb lean mass in this study are not likely to be driven directly by the acute elevations in serum hormone levels (West and Phillips 2012; Schoenfeld 2013; Egerman and Glass 2014), and differences between ISO and AEL may not have arisen directly from the observed differences in acute hormonal responses. However, it is interesting, and of potential practical importance to athletes and coaches, to identify whether acute hormonal responses might indicate the efficacy of a training stimulus to further induce improvements and adaptive changes (and how these responses possibly change throughout a period of training). On a group level, acute hormonal responses to the same strength training stimulus (described in this study as experimental loading) after a period of training in previously untrained subjects have been shown to be: (1) enhanced or prolonged in the case of total testosterone (T) (Ahtiainen et al. 2003; Walker et al. 2015), (2) suppressed in the case of cortisol (C) (Kraemer et al. 1999; Walker et al. 2015), and (3) unchanged or occasionally enhanced in the case of 22 kDa growth hormone (GH) (McCall et al. 1999; Izquierdo et al. 2009; Walker et al. 2015). Conversely, acute T and GH responses following short‐term training have been shown to be attenuated in already strength‐trained subjects (Ahtiainen et al. 2003; Mangine et al. 2015) and may account for different findings/associations between studies, although it must be remembered that few studies have examined these responses in already‐trained individuals.

Previous observations have shown a positive relationship between training‐induced adaptations in strength or muscle mass and maintained/larger acute hormonal responses (in T and GH, Ahtiainen et al. 2003; Walker et al. 2015, respectively) after training in previously untrained subjects. Based on these data, it has been proposed that the ability to continuously respond to a specific training stimulus could be observed in the magnitude of acute hormonal response to the same loading protocol when comparing responses before and after a period of training. In other words, a blunted hormonal response to the training stimulus may be an indication that the stimulus is no longer effective (i.e., it is sufficient but not excessive) to induce adaptation and the stimulus should be altered, such as observed in ISO. In this study, the AEL group maintained their acute hormonal response after 9 weeks of training along with greater improvements in strength and muscle mass during weeks 6–10 of training compared to ISO, which is in agreement with this hypothesis; this study is the first to demonstrate this relationship in already strength‐trained individuals.

Regarding GH and C concentrations, one possible explanation for the current findings is that elevations in circulating GH (Yarasheski et al. 1992; Wee et al. 2005) and C (Simmons et al. 1984) lead to metabolism of fatty and amino acids, respectively, to be used as sources of energy. It is possible, therefore, that the acute hormonal responses to training, such as ISO and AEL stimulate the liberation of energy to support demand during both loading and recovery, at least at the beginning of the training program. According to this hypothesis, only accentuated eccentric loading required, and triggered, a release of energy after the training period since the acute GH and C responses were maintained in AEL at week 9. Considering that muscular remodeling is an energy‐consuming process and may require prior breakdown, these findings may suggest a potential role of acute GH and C responses to loading‐ acute GH and C responses may exert an indirect influence on tissue remodeling following loading. This may explain why statistically significant and positive (albeit weak) relationships with the magnitude of hypertrophy have been previously observed (West and Phillips 2012) and partly explain the further increases in strength and hypertrophy during weeks 6–10 in AEL in the present study and in a larger cohort of subjects described previously (Walker et al. 2016).

Cortisol is also involved in regulation of the inflammatory response to exercise, and strength training has been shown to induce acute increases in regulatory cytokines (Izquierdo et al. 2009). One possibility for the lower C response in ISO and maintained response in AEL is that cortisol concentration is reflective of the pro‐ and anti‐inflammatory environment as a consequence of the different experimental loadings. For example, if isoinertial loading did not lead to a need for a robust inflammatory response during week 9, then an elevated C response may not be expected. This is, of course, speculation and would require follow‐up analyses investigating these potential causes specifically.

In the case of 22 kDa GH, it appears that some inferences may be drawn as to the efficacy of a training stimulus to induce improvements in strength and muscle mass (see correlation analyses in results section). Given that data were only obtained in 8 AEL subjects, it may have been expected that only a very large effect between these variables would yield a statistically significant correlation value (we observed an *r* value of 0.53–0.62, *P* = 0.11–0.16). Nevertheless, these moderate associations might suggest that a maintained, or larger, GH response at the end of a training period reflects a continued positive response to a training stimulus, while a lesser response may represent reduced potential for further adaptation to the current training stimulus. This hypothesis should be confirmed using a larger cohort of already strength‐trained subjects, perhaps with testing for longer than 15 min post‐loading given that acute GH and C responses may not peak until approximately 30 min post‐loading (e.g. Kraemer et al. 1999).

Further important considerations relate to the elements of strength training that influence the magnitude of acute hormonal response during experimental loadings. First, both groups demonstrated similar increases in volume load from week 2 to week 9 and there were no between‐group differences in volume load during either experimental loading test. This is an important point as the total work performed during loading has been shown to influence the acute hormonal response (Gotshalk et al. 1997). Consequently, the total work done does not appear to be a factor influencing the present results. Second, the level of anaerobic metabolism required to meet energy demand during loading has been shown to influence the acute growth hormonal responses (Gordon et al. 1994). While intramuscular lactate concentrations were not measured in the present study, the observation of similar blood lactate responses in ISO and AEL before and after training suggests that the energy derived from anaerobic metabolism was similar during the study; this conclusion assumes that buffering capacity/lactate shuttling/injection rate adaptations were not different between the groups. Hence, other stimuli may have influenced GH responses. One possibility is that the greater external load used during accentuated eccentric loading elicited greater motor unit activation during the eccentric phase (Ojasto and Häkkinen 2009). This greater activation has been shown to stimulate a greater hormone release from the pituitary gland (Ju 1999). In the context of the present study, it could be hypothesized that the greater muscle activation in AEL allowed for a continued GH response in the last 5 weeks of training (Ojasto and Häkkinen 2009), which may partly explain the greater increase in MVC (weeks 6–10) and larger GH response at week 9 when compared to ISO. This would further support the hypothesis that the observed hormonal responses were related to the homeostatic challenge of each loading protocol rather than being able to exert a direct influence on adaptation.

Another consideration is that subjects performed three sets of bilateral leg press followed by three sets of unilateral knee extension. While this may be considered typical of a medium‐intensity, high‐volume training program, it presents issues for the interpretation of acute hormonal responses. As observable in Figure 3, blood lactate and T concentrations began to decrease after mid‐loading. It has previously been established that multi‐joint exercises requiring use of a larger muscle mass (e.g., squat and leg press) elicit a greater hormonal response when compared to single‐joint exercises (e.g., knee extension) (Volek et al. 1997; Häkkinen et al. 1998). This may have influenced the potential magnitude of difference between ISO and AEL, and it is perhaps recommendable to perform future studies using a larger volume of multi‐joint exercise(s). Also, T has been shown to acutely elevate during medium‐intensity, high‐volume loading (Kraemer et al. 1990; Häkkinen and Pakarinen 1993) and this acute elevation has been recently shown to partly result from a reduced metabolic clearance rate rather than greater T production/secretion (Ahtiainen et al. 2015), as well as plasma volume shifts. The strength‐trained subjects in the study of Mangine et al. (2015) did not demonstrate significant elevations in T before or after training, while the blunted response observed in week 9 of ISO in the present study is in‐line with the findings of Ahtiainen et al. (2003).

As mentioned in the introduction, circulating hormone concentrations can be affected by both upstream events and downstream targets. For example, reduced production and secretion at the gland would lower hormone concentration if receptor activity remained constant, increased receptor activity and maintained production/secretion or both reduced production/secretion and increased receptor activity could also lower hormone concentrations. Since we did not measure upstream effectors of production/secretion (e.g., GH‐releasing hormone, adrenocorticotropic hormone, etc.) we cannot be certain of the cause of the observed blunted acute response in ISO. However, in our opinion, it is unlikely that there was upregulated receptor activity in ISO when studies show an acute downregulation of receptor activity immediately after training (e.g., Ratamess et al. 2005). Also, this type of adaptation would likely promote adaptation to the training stimulus and this was not observed in weeks 6–10 in ISO. It would, therefore, be of interest to determine the reasons for the present disparity between ISO and AEL groups in future studies.

This study was specifically designed to match the total number of sets and repetitions performed by both ISO and AEL during training (i.e., six sets of either 6‐RM or 10‐RM to concentric failure) in order to determine whether different magnitudes of adaptation could be observed whilst using an externally valid experimental design (i.e., it is unlikely that athletes would reduce volume significantly when using accentuated eccentric loading). It follows that AEL subjects performed greater volume load during the lowering phase of the leg press and knee extension exercises compared to ISO (as seen in Fig. 2). The subjects were matched for knee extensor strength and the subjects in AEL would have been expected to lift the same concentric load as ISO. Nevertheless, the accentuated eccentric load (+40%) negatively affected the subjects' abilities to perform the subsequent concentric lift and consequently reduced the total load lifted (i.e., concentrically) in AEL. Thus, there were no differences in total volume load between the groups when taking into account the loads lifted and lowered (Fig. 2). While training volume (or total work) is an important factor influencing the magnitude of training‐induced improvements (Wernbom et al. 2007), the equivalent volume loads imposed in the present study suggest that this element of strength training cannot account for the continued improvement in AEL from mid‐ to post‐training.

In conclusion, acute hormonal responses were maintained in AEL after 9 weeks of training in already strength‐trained men, whereas, training using traditional isoinertial loading was not sufficient to maintain such a response. These findings, when set against the greater improvement in strength and lower‐limb lean mass in AEL during the last 5 weeks of training, suggests that trained individuals who (primarily) perform medium‐load, high‐volume strength training may derive a benefit from training with accentuated eccentric loads in the longer term. The data are consistent with the hypothesis that acute hormonal responses might reflect a continued response to the training stimulus. However, since no significant relationships were observed between the changes in acute hormonal responses and the training‐induced improvements, the tracking of acute hormonal responses may not provide a sensitive or reliable basis on which to determine the efficacy of a training stimulus.

## Conflict of Interest

The authors declare no conflict of interest.

## Data Accessibility
